# Design, Implementation, and Validation of a Pulsatile Heart Phantom Pump

**DOI:** 10.1007/s10278-020-00375-5

**Published:** 2020-08-10

**Authors:** Volkan Tuncay, Jan Zijlstra, Matthijs Oudkerk, Peter M. A van Ooijen

**Affiliations:** 1 Center for Medical Imaging - North East Netherlands (CMI-NEN), University of Groningen, University Medical Center Groningen, PO BOX 30001, NL-9700 RB Groningen, Netherlands; 2 Institute of Engineering, Hanze University of Applied Sciences, Groningen, Netherlands; 3 Department of Radiation Oncology, University of Groningen, University Medical Center Groningen, Groningen, Netherlands

**Keywords:** Pulsatile pump, Phantom design, Cardiovascular circulation mimicking, Computed Tomography imaging, Pulsatile flow

## Abstract

The developments in Computed Tomography (CT) and Magnetic Resonance allow visualization of blood flow in vivo using these techniques. However, validation tests are needed to determine a gold standard. For the validation tests, controllable systems that can generate pulsatile flow are needed. In this study, we aimed to develop an affordable pulsatile pump and an artificial circulatory system to simulate the blood flow for validation purposes. Initially, the prerequisites for the phantom were pulsating flow output equal to that of the human cardiac pulse pattern; the flow pattern of the mimicked cardiac output should be equal to that of a human, a variable stroke volume (40–120 ml/beat), and a variable heart rate (60–170 bpm). The developed phantom setup was tested with CT scanner. A washout profile was created based on the image intensity of the selected slice. The test was successful for a heart rate of 70 bpm and a stroke volume of 68 ml, but the system failed to work at various heartbeats and stroke volumes. This was due to the problems with software of the microcontroller. As conclusion in this study, we present a proof of concept for a pulsatile heart phantom pump that can be used in validation tests.

## Background

The advent of new and improved scanning algorithms for both Computed Tomography (CT) and Magnetic Resonance Imaging (MRI) combined with their increased temporal resolution have facilitated the ability to measure and visualize blood flow and perfusion in vivo using these techniques [1, 2]. However, accurate validation is needed to determine the gold standard. Phantoms are commonly used for the validation purposes [3, 4]. For proper validation and calibration, a controllable system is required which mimics the dynamic pumping function of the heart.

Phantoms are developed using different kinds of pumps to mimic the blood flow [5–7]. However, the type of pump used has major impact on its applicability. Laminar flow pumps do not generate a pulsatile flow, which is needed to mimic the blood flow through the cardiovascular system [8, 9]. When pulsatile flow is needed, most of the time commercial pulsatile pumps are used [7, 10, 11].

In this study, we aimed to develop an affordable pulsatile pump and a mock circulatory system in order to simulate the blood flow for validation tests and other development purposes such as image processing and medical device testing. The prerequisites for an ideal phantom are identified as follows:

1. Controllable pulsating flow output equal to the human pulse pattern.
2. The flow pattern of the mimicked cardiac output should be equal to that of a human.
3. Stroke volume variable between 40 and 120 ml/beat.
4. Heart rate variable between 60 and 170 bpm.

The developed phantom was tested with a CT scanner.

## Methods

### The Setup

**Fig. 1:**
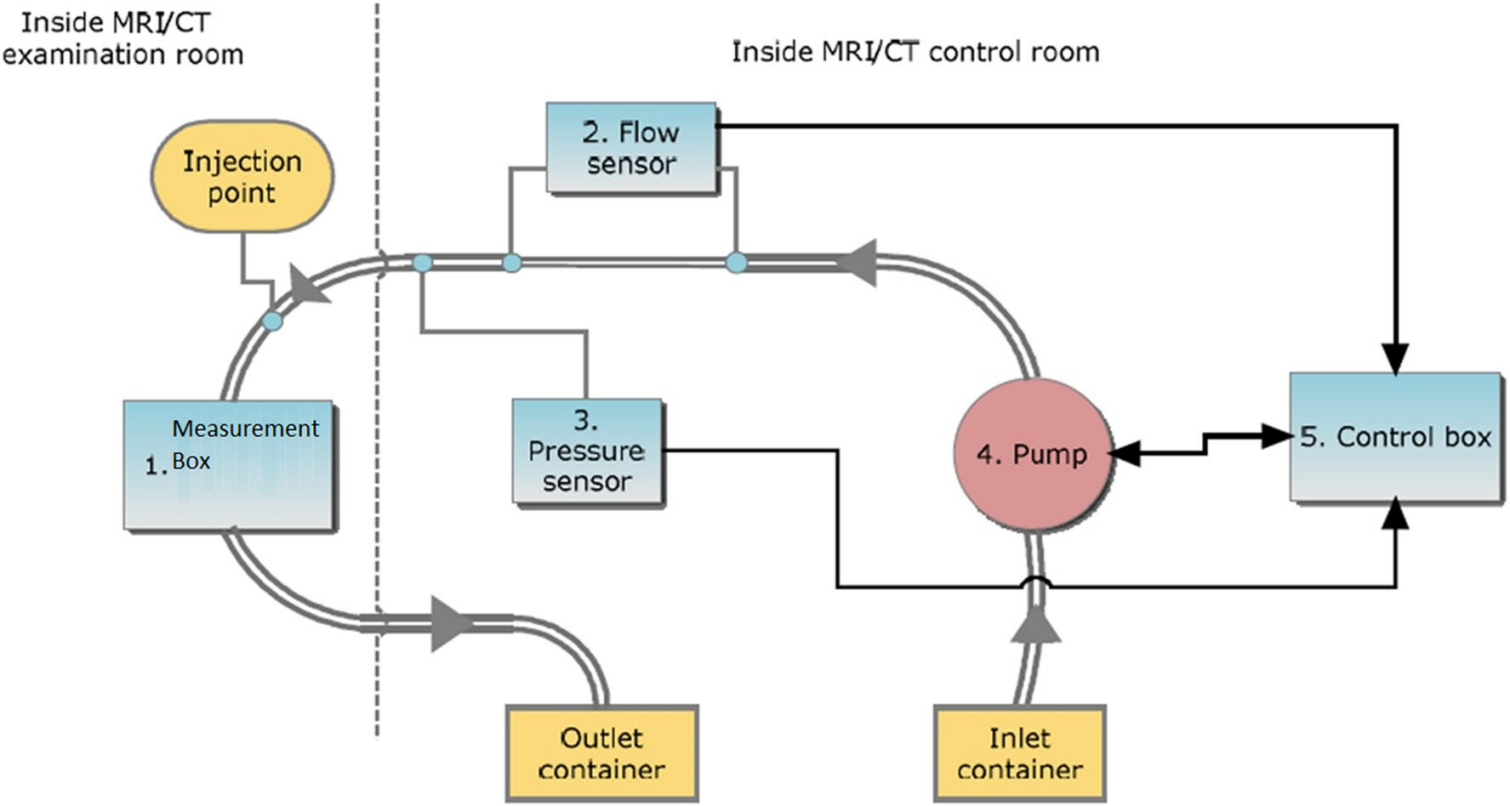
Flowchart for the pulsatile heart phantom

The main components of the phantom setup are the measurement box, the pump, the control box, the pressure, and the flow sensors. These components are connected together using tubing (Fig. 1). The phantom pump should create a pulsatile flow through the measurement box. The measurement box has an in- and outflow and can contain different vessel/valve configurations or other phantoms. The measurement box is the part of the phantom setup that will be placed inside the bore of the CT or MRI and thus should not contain any ferro-magnetic parts. Pump and measurement system should allow use of blood as liquid as well.

### The Pump

**Fig. 2:**
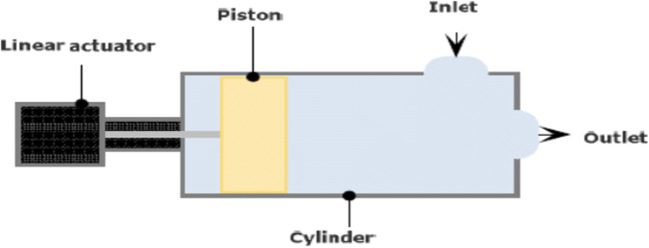
The pulsatile pump

The pump is a piston pump using a linear actuator (L4118L1804-T5X5A50, Nano-Tec) to drive the piston inside a cylindrical chamber. The linear actuator has a fixed step size of 0.025 mm. The movement of the actuator will result in a pulsatile, one-directional flow, by placing one-way valves at the inlet and outlet (Fig. 2).

The cylinder is from one block of aluminum with a cavity diameter of 59.9 mm and a length of 101.0 mm resulting in a maximum volume of the cylinder of 284.6 ml. Because of the size of the piston, the limitations of the length of the axis of the actuator, and the general construction of the pump, the actual working maximum stroke volume of the pump is 110 ml.

The speed of the piston is dependent on the heart rate, stroke volume, and flow pattern set using the control panel. The volume/step is constant at 0.0704 ml, which defines the amount of volume displaced with one-step movement of the linear actuator. Using the settings and this constant, the speed of the linear actuator (*V*) is determined using the following formula:

$$ V\left[ mm/\mathit{\sec}\right]=\left(\frac{Stroke\ Volume\left[ ml\right]}{Volume/ step\left[ ml\right]}\ast 0.025\right)/\left(\frac{60}{Heart\ Rate\left[ bpm\right]}\ast 1/2\right) $$

### The Control Box

The control box houses all the electronic parts, such as the microcontroller for controlling the system and an interface for operating the system. The microcontroller (Atmel, AVR 32 Bits AT32UC3C1512) connects all the parts of the hardware and ensures the proper operation of the setup.

A rotary encoder is used to navigate through the menu of the control box. This rotary encoder allows selection by rotation and acknowledgement of a choice by pushing on the rotary encoder. The pump is activated by a start button. The stop button is a locking button, so once pressed it needs to be released by pressing on it again to activate the pump again. This prevents the rapid start and stop of the pump, which might lead to damaging the pump. Unauthorized usage is prevented by integration of a key switch. Feedback to the user is provided by a four-line, twenty character Liquid Crystal Display (LCD). This display always provides the state of the system on the first line (e.g., start the pump or change heart rate setting). The second and third line shows additional information, such as selected heart rate or values acquired from the sensors. The fourth line shows the possible action (e.g., menu scroll by “<” and “>”). When a variable is surrounded with “<” and “>,” the value of the variable can be adjusted by turning the rotary encoder.

The correct operation of the stepper motor is checked using position feedback with a 10-turn potentiometer on the axis of the stepper motor. This suffices since the motor axis rotates a maximum of 9.5 turns to fully expand. By measuring the voltage of the potentiometer, the exact position of the piston can be derived. This feedback will not be used to create a feedback loop to adjust the cardiac output. But it can be used to monitor if the piston is stuck or that the piston is not attached to the axle any more. It is also convenient to use during development of controlling the pump.

The trigger pulse used for synchronizing the pump action with the acquisition of the CT or MR scanner is a logic signal between 0.0 and 5.0 VDC. The frequency of the pulse should be equal to the set heart rate. When the pulse is logic low, the pump is filled with liquid, which represents the diastolic phase. When the logic level of the pulse is high, the liquid is pumped out, which represents the systolic phase. The transition from a low level to a high level rising edge indicates the beginning of the QRS complex.

The hardware of the trigger consists of an op-amp configured as a differentiator and comparator. The trigger circuit uses the signal from the position sensor. When this signal rises, the liquid is pumped out, and while the signal falls, liquid is sucked into the pump. The signal from the position feedback is connected to the differentiator and is used to create a pulse. Since the amplitude of the block signal is not between 0.0 and 5.0VDC, a comparator is used.

### Software Design

The program is designed with a main loop or a menu structure. In this loop it is possible to switch between states by rotating the rotary encoder and select the desired state through pushing on the rotary encoder. Each of the states in the menu allows the user to control the functions of the system. For error handling every state refers to the error state in case of error. The error state disables the pump and lets the user know what kind of error took place such as turned off key switch voltage drop below the required value.

When the system starts up, the program starts with state zero. In this state all the settings are configured, and the condition of the system is tested. If there are no problems, the program advances to state one. In state one it is possible to start the pump with the start button. The state is changed into state five, and the pump is initialized for correct functioning before the pumping sequence starts. During the pumping action, the sensors sample and send the acquired data to a computer. This communication is done via a USB connection. The user needs to press on the stop button in order to leave the state and to stop the pumping action. Then the system goes back to the first state.

When the program is back in the main loop, it is possible to change the heart rate and stroke volume. The desired state needs to be selected by turning the rotary encoder. When the display shows the desired state, it can be selected by pushing on the rotary encoder. Then the set value can be adjusted again by rotating the rotary encoder, and it can be saved by pushing on the rotary encoder. The options state allows the user to read the values of the sensor without the pumping action.

### Validation Test

**Fig. 3:**
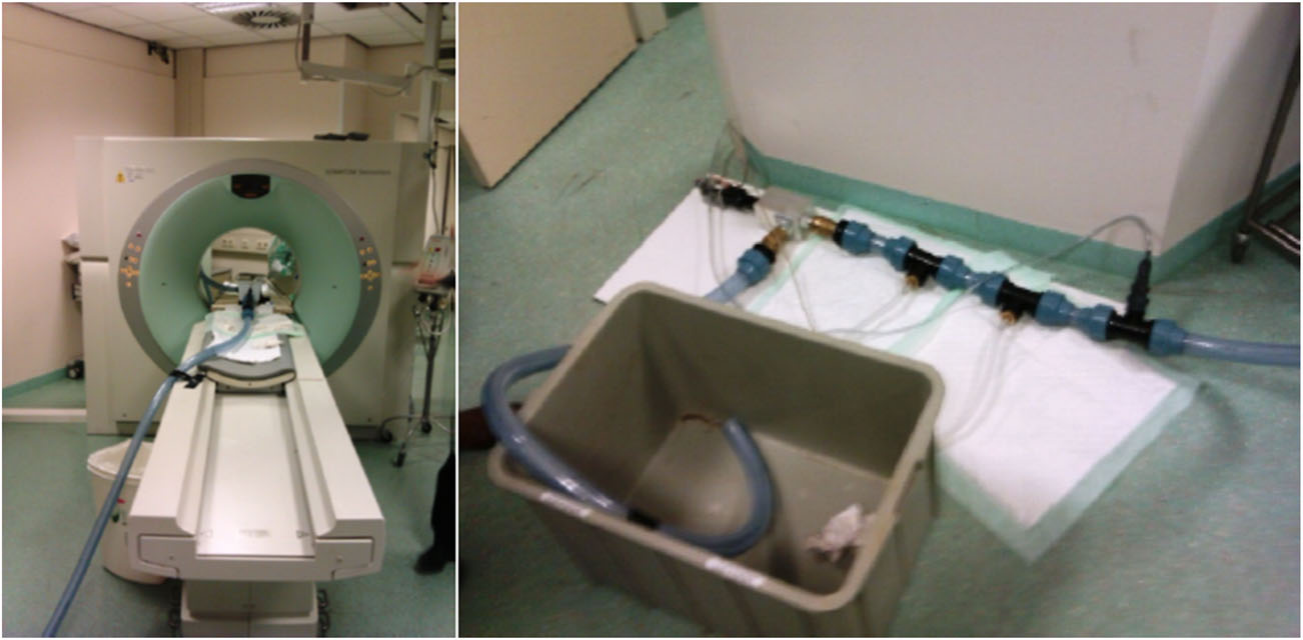
The testing with Computed Tomography

The phantom setup was tested with a CT scanner in order to validate the pulsatile heart phantom (PHP) (Fig. 3). During the test, water was continuously pumped through the tubes with a heart rate of 70 bpm and a stroke volume of 68 ml. Approximately 20 cm before the measurement box, contrast liquid was pumped in the tubing. A section of the tubing is scanned with the CT to create the intensity profile of the washout.

## Results

### Validation Test Results

**Fig. 4:**
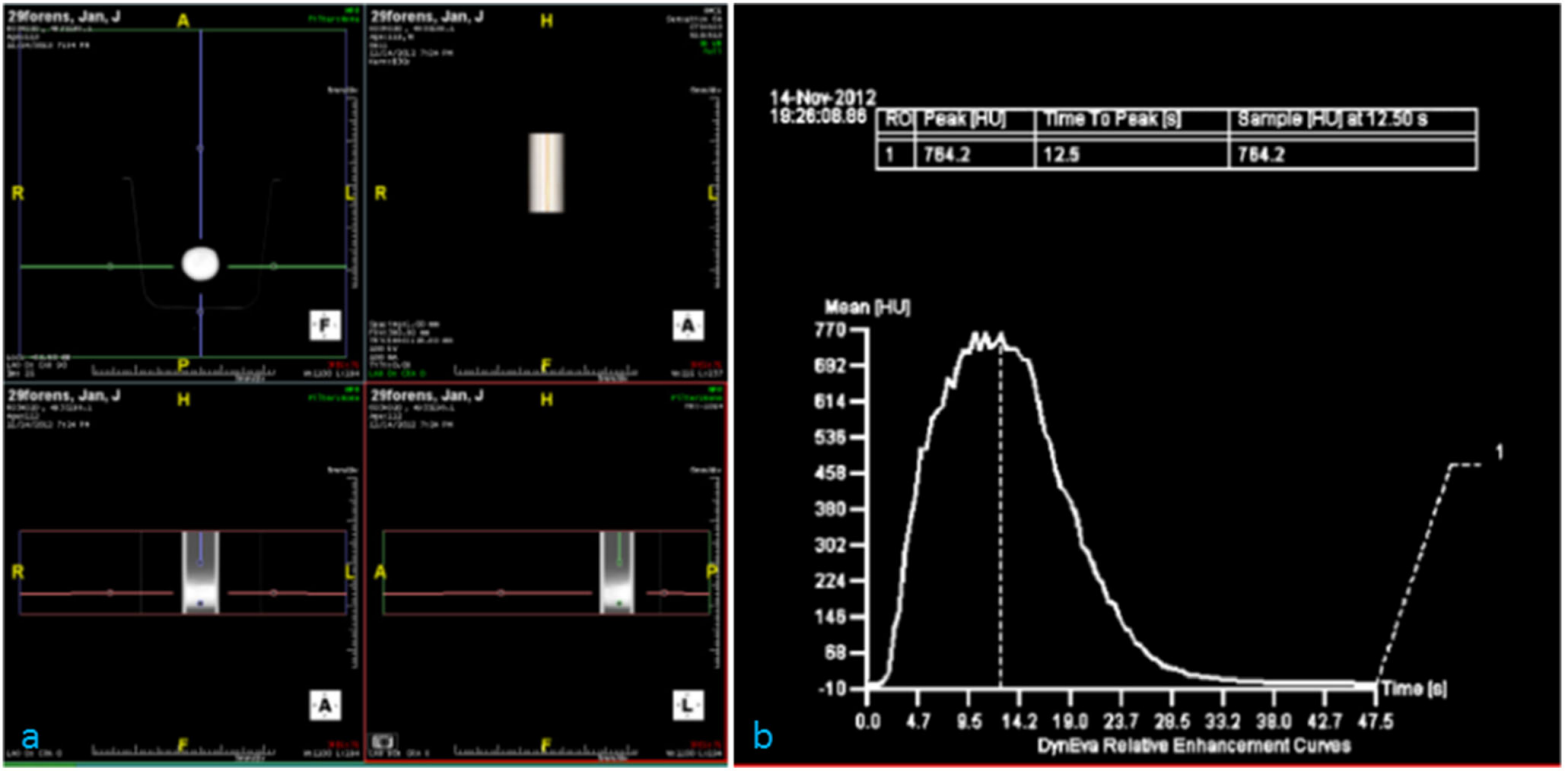
Result of the test with Computed Tomography. The 3D image of the scanned section of tubing (**a**) and the washout profile (**b**)

The 3D image and the washout profile acquired in the validation test scan are given in Fig. 4. The test was successful for the given heart rate and stroke volume. It showed that the designed pump can create pulsatile flow and the PHP can be used to simulate the blood circulation in the circulatory system. However, the goal of adjusting the heart rate and the stroke volume could not be achieved. It was caused by the software of the microcontroller.

## Discussion

The developed pump for the PHP can create pulsatile flow, and the PHP can be used to mimic the blood circulation. The pump was connected via tubes to the measurement box, which is placed in the gantry of the scanner. In the measurement box, various types of medical devices such as heart valves or stents can be placed to conduct quality or development tests.

A preliminary test was done to test the principle of the PHP system. It was possible to validate the measured flow in the CT to the manually measured flow rate in the PHP. Quite some improvements of the PHP came up during the test. The two most important modifications were the setup of the flow sensor and the driver, which controls the pump.

There are several studies reporting the use of pulsatile pump in mock-up settings. Hiai et al. compared the diagnostic performance of CTA and MRA for intracranial aneurysm evaluation using commercially available pulsatile pump [12]. In a more recent study, Shephard et al. developed patient-specific coronary artery phantoms using 3D printing and commercially available pulsatile pump. The phantoms were used for software validation [13]. The study most similar to this study is conducted by Youn et al. They developed a phantom setup and pulsatile pump specifically to investigate use of artificial neural network to develop a contrast-enhanced computed tomography angiographic protocol based on ideal bolus geometry [14]. Hiai et al. and Shephard et al. did not develop their own pump but integrated commercially available pumps into their setup. Youn et al. did develop their own pulsatile pump. However, the focus of their study was not to develop a multi-purpose phantom but to build a phantom to validate a specific software and to mimic a specific region of interest. On the other hand, in this study a multi-purpose phantom with indigenous pump that can host various vessel and prosthesis configurations was developed.

The issue with the setup of the flow sensor was that it was located in a shunt tube. The shunt tube had a high resistance therefore; hardly any liquid flowed through the shunt tube. To solve this issue, a differential pressure sensor can be used to measure the pressure over a shunt tube with fixed hydraulic resistance. The flow can be derived from the measured pressure drop. A pressure sensor is used because the response time of the flow sensor was too slow to measure the flow pattern accurately as function of time.

The issue with the driver was that the signal required for the driver was created by the microcontroller. In the same time, the microcontroller had to drive the pump simultaneously. This resulted in wrong values and incorrect driver signals. To solve this issue a separate microcontroller can be used to drive the pump independent from the main microcontroller.

## Conclusion

A principle of proof is given for a heart phantom pump to adjust and measure dynamic flow as a validation tool for flow measurement in MRI and CT. This is confirmed with a test in a CT. However, a significant a number of improvements should be incorporated to let the PHP operate as initially specified. With these improvements, the PHP will be a challenging tool for validation of flow in imaging modalities and might offer opportunities in other disciplines as well (i.e., source for artificial circulation systems).
